# A Single High-Sensitivity Cardiac Troponin T Strategy for Ruling Out Myocardial Infarction

**DOI:** 10.1155/2024/2241528

**Published:** 2024-03-25

**Authors:** Patrik Gilje, Moman A. Mohammad, Andreas Roos, Ulf Ekelund, Jonas Björk, Bertil Lindahl, Martin Holzmann, Arash Mokhtari

**Affiliations:** ^1^Lund University, Skåne University Hospital, Department of Cardiology, Lund, Sweden; ^2^Department of Medicine, Karolinska Institute, Solna, Stockholm, Sweden; ^3^Department of Emergency and Reparative Medicine, Karolinska University Hospital, Huddinge, Stockholm, Sweden; ^4^Lund University, Skåne University Hospital, Department of Internal and Emergency Medicine, Lund, Sweden; ^5^Occupational and Environmental Medicine, Lund University, Lund, Sweden; ^6^Department of Medical Sciences and Uppsala Clinical Research Center, Uppsala University, Uppsala, Sweden

## Abstract

**Background:**

Ruling out acute myocardial infarction (AMI) in the emergency department (ED) is challenging. Studies have shown that a high-sensitivity cardiac troponin T (hs-cTnT) <5 ng/L or <6 ng/L at presentation (0 h) can be used to rule out AMI. The objective of this study was to identify whether an even higher hs-cTnT threshold can be used for a safe rule out of AMI in the ED.

**Methods:**

The derivation cohort consisted of 24,973 ED patients with a primary complaint of chest pain. In this cohort, we identified the highest concentration of 0 h hs-cTnT that corresponded to a negative predictive value (NPV) of ≥99.5% for the primary endpoint of AMI/all-cause death within 30 days and the secondary endpoint of all-cause death within one year. The results were validated in two cohorts consisting of 132,021 and 1167 ED chest pain patients.

**Results:**

The 0 h hs-cTnT threshold corresponding to a NPV of ≥99.5% for the primary endpoint was <9 ng/L (NPV: 99.6% and 95% CI: 99.5–99.7). This cutoff provided a sensitivity of 96.2% (95% CI: 95.2–97.1) and identified 59.7% of the patients as low risk compared to 35.8% and 43.9% with a 0 h hs-cTnT <5 ng/L and <6 ng/L, respectively. The results were similar in the validation cohorts and seemed to perform even better in patients where the 0 h hs-cTnT was measured >3 h after symptom onset and in those with a nonischemic ECG and nonhigh risk history.

**Conclusions:**

A 0 h hs-cTnT cutoff of <9 ng/L safely rules out AMI/death within 30 days in a majority of chest pain patients and is a more effective strategy than the currently recommended <5 ng/L and <6 ng/L cutoffs. This trial is registered with NCT03421873.

## 1. Introduction

Chest pain is one of the most common presenting complaints at the emergency department (ED), constituting about 5% of all ED visits [1]. The management is driven by ruling-in patients with serious conditions such as acute myocardial infarction (AMI) and at the same time identifying low-risk patients suitable for early discharge. However, only a small minority of patients has an AMI and a large proportion of patients are either admitted or undergo further investigations with serial troponins in the ED [2]. Developing a more efficient management of these patients could thereby have a large impact.

The use of high-sensitivity troponins (hs-cTnT) has enabled the use of more rapid rule-out strategies [3–5]. Currently, the European Society of Cardiology (ESC) guidelines state that a hs-cTnT <5 ng/L at presentation (0 h) can be used to rule out AMI [6]. This cutoff, which represents the limit of detection (LoD) of the assay, has been shown in several studies to perform well [7, 8]. However, the US Food and Drug Administration has only approved reporting of hs-cTnT down to a concentration of 6 ng/L, as this constitutes the limit of quantification (LoQ) of the assay. Using a 0 h hs-cTnT <6 ng/L has also been shown to safely rule out AMI [9]. Using an hs-cTnI assay, the optimal 0 h cutoff for safe and efficient AMI rule out is higher than both the LoD and the LoQ [10], but to our knowledge, no studies have evaluated this approach with the hs-cTnT assay.

The objective of this study was to identify whether a higher hs-cTnT threshold than the LoD and the LoQ can safely rule out AMI or all-cause death within 30 days in ED chest pain patients and to validate the results in a geographically separate cohort.

## 2. Methods

### 2.1. Study Design and Participants

The derivation cohort consisted of patients included in a multicentre implementation study with registry-based follow-up (ESC-TROP: Effectiveness and Safety of the European Society of Cardiology 0-/1-h Troponin Rule-Out Protocol; NCT03421873), and the methods have been described in detail elsewhere [11]. In brief, we included all patients presenting to one of five EDs in southern Sweden with a primary complaint of chest pain of nontraumatic origin between the 1^st^ of February and the 30^th^ of November 2017 and between the 1^st^ of February and the 30^th^ of November 2018. We excluded patients with (1) a final diagnosis of ST-elevation myocardial infarction (STEMI) during the index visit, (2) patients in whom no hs-cTnT was ordered at the ED visit, (3) patients leaving the ED against medical advice, (4) patients without Swedish personal identity number in whom the follow-up was not possible through national registries, (5) patients with hemolysis in the 0 h hs-cTnT sample defined as a H-index ≥100 (the level recommended by the manufacturer), and (6) patients who actively declined participation. This study received ethical approval from the Regional Ethics Review Board in Lund without the need for written informed consent; patients could, however, withdraw from participation at any time, without specific reason by contacting the study administration.

### 2.2. Validation Cohorts

The validation cohort 1 consisted of patients >18 years of age with a first visit to the ED at nine different hospitals in Stockholm and Gothenburg, Sweden, from May 1, 2012, through December 31, 2016, with a chief complaint of chest pain and at least one hs-cTnT test analysed concurrently. All patients without a final diagnosis of STEMI associated with the visit were included. The study protocol was approved by the Regional Ethics Review Board of Stockholm, and no written informed consent was required.

Validation cohort 2 was a prospective observational study, and the methods have been described in detail previously [12]. Patients presenting to the ED of Skåne University Hospital in Lund with a primary complaint of nontraumatic chest pain and for whom hs-cTnT was ordered at presentation enrolled between February 2013 and April 2014 were included. This study did not enroll those with severe communication barriers and patients with STEMI. Patients with missing data and those with 0 h samples with hemolysis (H-index ≥100) were excluded. The study was approved by the regional Ethical Review Board in Lund and all patients provided written informed consent.

### 2.3. Data Collection and Outcomes

Patients in the derivation cohort were identified through electronic ED patient logs and all patients with a nontraumatic chest pain were enrolled by default. Data management and coordination were performed by Clinical Studies Sweden Forum South, which is an independent research organization under the Swedish government. Laboratory data were obtained from each hospital laboratory database. The troponin assay used in all hospitals was the Elecsys hs-cTnT assay (Roche Diagnostics), and samples were analysed in real time and used for clinical decision making. This assay has a limit of detection of 5 ng/L, a limit of quantitation of 6 ng/l, and a coefficient of variation <10% at the 99th percentile of 14 ng/L [13]. Patients were managed at the discretion of the treating physician. Data on patient comorbidities and current medications were obtained from regional electronic medical records systems which includes all hospitals as well as primary care in the region, the National Patient Register, and the Swedish Prescribed Drug Register [14, 15]. Data collection methods for the validation cohort 1 and a description of the different centers in the cohorts are presented in the supplemental material, appendix 1 and 2. In validation cohort 2, data were prospectively collected by research assistants using a study form [12].

The primary outcome was AMI or all-cause death within 30 days (including the index visit), and the secondary outcome was all-cause death at 1 year.

The ESC-TROP trial utilized a registry-based follow-up, and AMI was based on a diagnosis in the SWEDEHEART registry [16]. As not to miss patients potentially not included in SWEDEHEART, diagnoses were also obtained from the regional electronic health records from all hospitals in the region. As not to miss patients who potentially sought care outside of our region during the 30-day follow-up, data were also obtained from the National Patient Register. Both SWEDEHEART and the National Patient Register are national registries that provide nationwide coverage of AMI events [14, 16]. Finally, to prevent misclassification of patients potentially missed during the index visit, potential events in patients discharged from the ED during the index visit were adjudicated by two independent cardiologists using the fourth universal definition of myocardial infarction (see supplemental material for further details) [17]. In case of disagreement, cases were reviewed by an adjudication committee and resolved by majority vote. Data on deaths and dates of death were obtained from the Swedish population register providing complete nationwide coverage [18]. Data on patients who migrated to other countries during the 30-day follow-up were also obtained from the Swedish population register, and these patients were considered lost to follow-up.

Details on definitions of outcomes for the validation cohort are provided in the supplementary material. In validation cohort 1, the diagnoses were also registry-based, while in validation cohort 2, all diagnoses were adjudicated by 2 independent cardiologists.

### 2.4. Statistical Analyses

Baseline characteristics are presented as medians and interquartile range for continuous variables, and categorical variables are displayed as counts and percentages.

Negative predictive values (NPV) with 95% confidence interval (CI) were calculated for the primary outcome across hs-cTnT cutoff concentrations, starting from <5 ng/L to the upper reference limit of 14 ng/L. We applied a safety cutoff of a NPV ≥99.5% as a prespecified requirement for the primary outcome since this is commonly regarded as an acceptable threshold [10, 19]. The highest 0 h hs-cTnT-value with ≥99.5% NPV was then tested in the validation cohort.

To ensure a NPV >99% with an expected value of 99.5%, a power calculation resulted in a required sample size of 2500 patients in the derivation cohort and an equal number of patients in the validation and validation cohorts each to attain an 80% statistical power with an alpha risk of 0.05.

The 0 h hs-cTnT threshold corresponding to a NPV of ≥99.5% was analysed in the following prespecified subgroups: age, sex, glomerular filtration rate (GFR) <60 vs. >60 mL/min/1.73 m^2^, history of AMI, diabetes mellitus, and time from ED admission to blood sampling (<1 h vs. ≥1 h). Cumulative incidence of the secondary outcome 1-year all-cause mortality was estimated using the Kaplan–Meier method for the derivation cohort and validation cohort 1 only since validation cohort 2 did not have a 1-year follow-up. All statistical analyses were performed using STATA MP version 16.1 for Macintosh (StataCorp, Texas, USA).

## 3. Results

### 3.1. Baseline Characteristics

**Figure 1:**
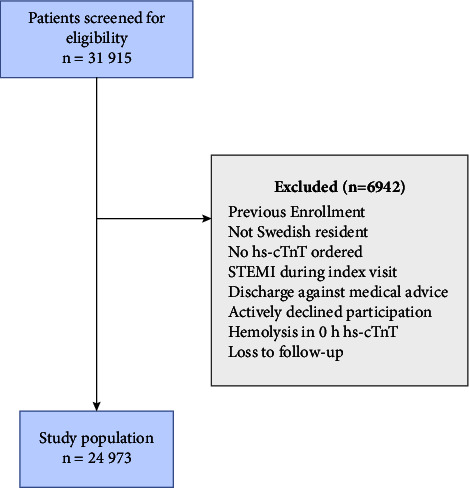
Patient flow. The flow diagram depicts number of patients included and excluded. hs-cTnT: high-sensitivity cardiac troponin T; STEMI: ST-elevation myocardial infarction.

After the application of the exclusion criteria, 24,973 ED patients were enrolled in the derivation cohort (Figure 1) and 132,021 in validation cohort 1 and 1167 in validation cohort 2. Patient characteristics of the three cohorts are presented in Table 1. The median age was 61 years in the derivation cohort, and 48% of the included patients were women. A history of coronary artery disease or diabetes mellitus was seen in 15.7% and 14.2%, respectively. The patients in the derivation cohort were somewhat older (median age 61 vs. 57 years) and had overall more cardiovascular risk factors/comorbidities than in validation cohort 1 but less cardiovascular risk factors than patients in validation cohort 2. A total of 1668 (6.7%) patients had an AMI/death event within 30 days in the derivation cohort, 7668 (5.8%) in validation cohort 1, and 88 (7.7%) in validation cohort 2. AMI within 30 days occurred in 1458 (5.8%) patients in the derivation cohort, 6924 (5.2%) in validation cohort 1, and 89 (7.6%) in validation cohort 2.

### 3.2. Primary Outcome

**Figure 2:**
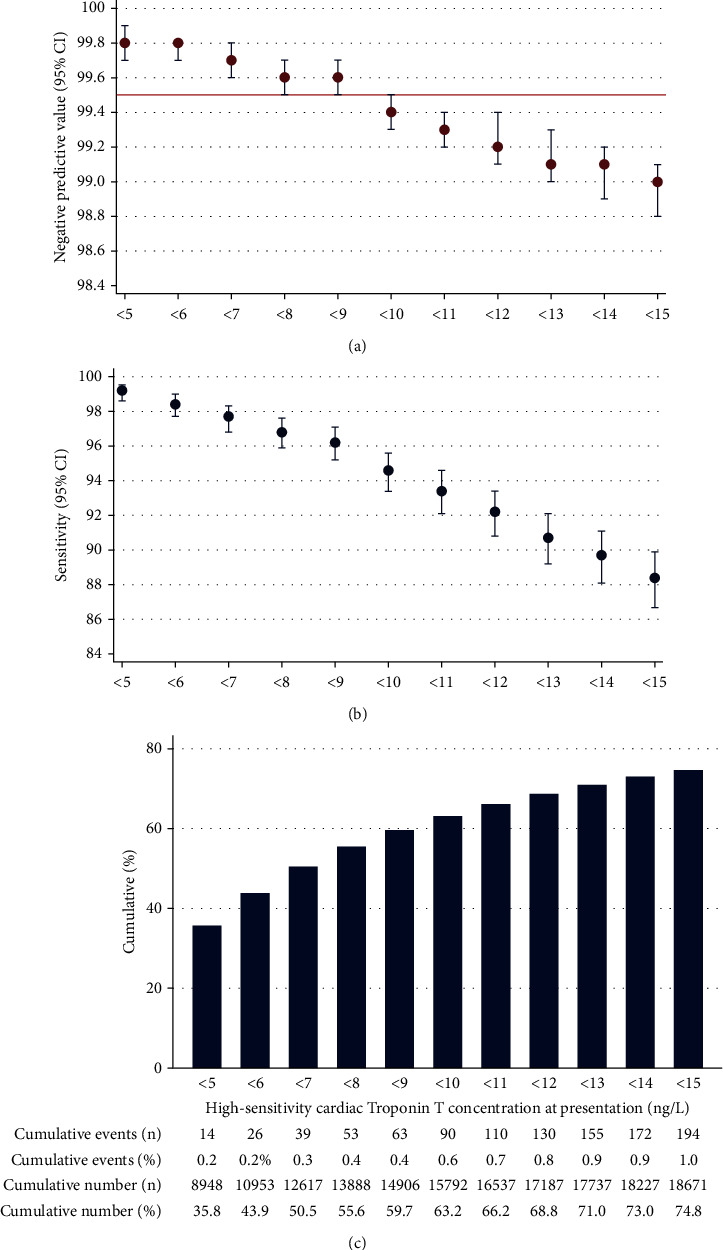
0 h hs-cTnT concentrations at presentation. (a-b) 0 h hs-cTnT and the negative predictive value/sensitivity of acute myocardial infarction and death within 30 days. (c) Cumulative proportion of chest pain patients in the emergency department with 0 h hs-cTnT below each threshold.

**Figure 3:**
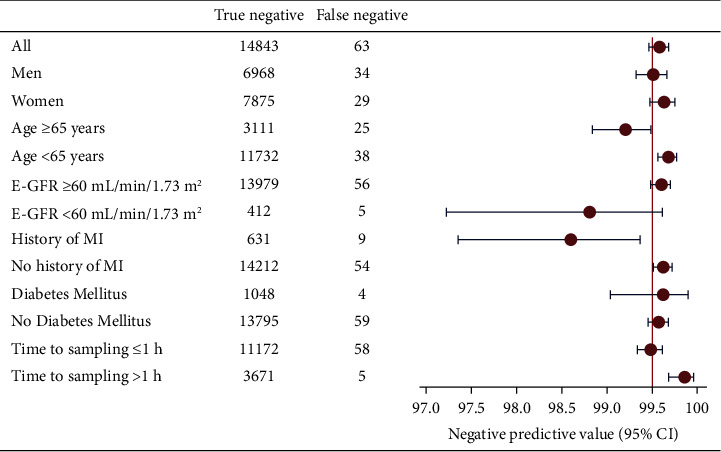
Subgroup analyses. Negative predictive value of 0 h hs-cTnT <9 ng/L for myocardial infarction or death within 30 days, stratified according to subgroup. E-GFR: estimated glomerular filtration rate (MDRD 4); MI: myocardial infarction.

The highest 0 h hs-cTnT threshold with a NPV of ≥99.5% for the primary outcome was <9 ng/L which identified 14906 patients (59.7%) for rule out with a NPV of 99.6% (95% CI: 99.5–99.7), sensitivity 96.2% (95% CI: 95.2–97.1), and negative likelihood ratio (LR-) of 0.06 (95% CI: 0.05–0.08) (Figure 2). Of the missed events, 57 were AMI and 6 were deaths. The LoD strategy of a 0 h hs-cTnT <5 ng/L identified 8948 patients (35.8%) for rule out with a NPV of 99.8% (95% CI: 99.7–99.9), missing 13 patients with an AMI and 1 death. The LoQ strategy of <6 ng/L had a similar NPV (NPV: 99.8% and 95% CI: 99.7–99.8) but classified more patients (43.9%) as rule out, missing 23 patients with an AMI and 3 deaths. Using a 0 h-cTnT <9 ng/L instead of <5 ng/L would thus have enabled rule out in an additional 5958 patients (23.9%), at the expense of a decrease in NPV from 99.8% to 99.6%. Only a 0 h hs-cTnT <5 ng/L had a sensitivity of >99%. NPVs; the sensitivity and LRs for the different cutoffs are provided in the supplemental material. The 0 h hs-cTnT of <9 ng/L strategy performed well (NPV ≥99.5%) in all subgroups except in patients with a history of AMI where the NPV was 98.6% (95% CI: 97.3–99.4; Figure 3). The NPV for those with a GFR <60 and those ≥65 years were also lower, but the CI included 99.5%.

### 3.3. Secondary Outcome

**Figure 4:**
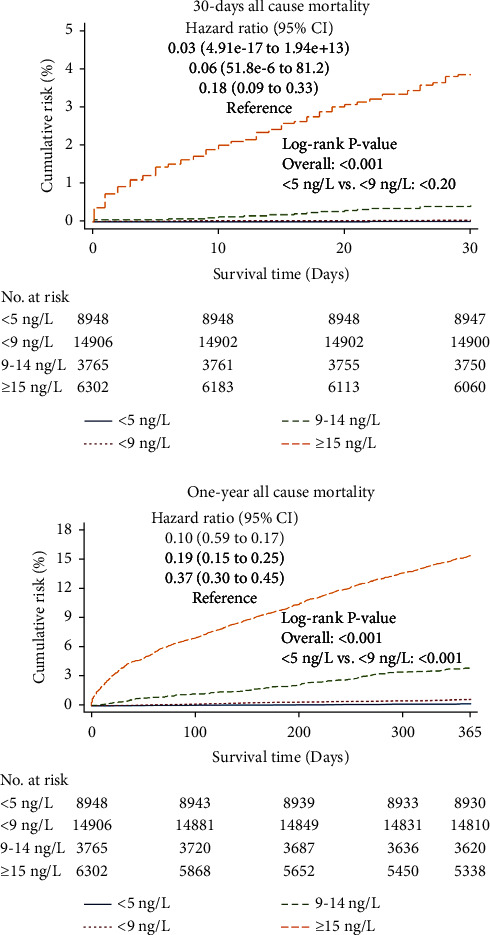
30-day and 1-year all-cause mortality. Cumulative incidence of 30-day and 1-year all-cause mortality in patients stratified according to 0 h hs-cTnT concentrations.

The overall 1-year mortality was 4.8%. The Kaplan–Meier curves for the secondary endpoints are shown in Figure 4. The 1-year mortality was 0.7% in patients with a 0 h hs-cTnT <9 ng/L. This was marginally higher than for 0 h hs-cTnT <5 (0.2%) and clearly lower than the 9–14 ng/L group (3.9%). The 30-day mortality with a 0 h hs-cTnT <9 ng/L was 0.04% compared to 0.03% and 0.01% for <6 ng/L and <5 ng/L, respectively.

### 3.4. Validation Cohort 1

A total of 87,762 patients (66.5%) in the validation cohort had a 0 h hs-cTnT of <9 ng/L. The application of this threshold resulted in a NPV for 30-day AMI/all-cause mortality of 99.6% (95% CI: 99.6–99.7), with a sensitivity of 95.8% (95% CI: 95.4–96.3) and LR- of 0.06 (95% CI: 0.05–0.07). The 1-year all-cause mortality among these patients was 0.6% (figure in supplemental material, appendix 5).

### 3.5. Validation Cohort 2

In this cohort, 669 patients (57.3%) had a hs-cTnT <9 ng/L which had a NPV of 99.4% (95% CI: 98.5–99.8), a sensitivity of 95.7 (95% CI: 89.4–98.8), and a LR- of 0.07 (95% CI: 0.03–0.18) (supplemental material, appendix 6). Among patients where 0 h hs-cTnT was measured ≤3 h from symptom onset, NPV was lower (98.5%) while among those with a measurement after >3 h, the NPV was 99.8% (95% CI: 98.7–100) with a sensitivity of 98.1% (95% CI: 89.9–100). The NPV and sensitivity were also higher among those who also had a nonischemic ECG (NPV: 99.5% and sensitivity: 96.6%) or a nonischemic ECG and a nonhigh risk history (NPV: 99.8% and sensitivity: 98.9%). The NPV was highest in patients who had a 0 h hs-cTnT measured after >3 h after symptom onset and a nonischemic ECG and a nonhigh risk history (NPV: 100% and sensitivity: 100%).

In the pooled analysis of the derivation and the validation cohorts, a 0 h hs-cTnT <9 ng/L yielded a NPV of 99.6% (95% CI: 99.6–99.7), a sensitivity of 95.9% (95% CI: 95.5–96.3), and a LR- of 0.06 (95% CI: 0.05–0.07) for the primary outcome.

## 4. Discussion

In three large cohorts of consecutive patients at multiple sites, we derived and validated a novel 0 h hs-cTnT cutoff which could enable a safe and rapid rule out of AMI in a large proportion of ED chest pain patients.

Hs-cTnT is essential in risk-stratifying patients with chest pain in the ED. The widely accepted Hs-cTnT LoD 0 h < 5 ng/L approach has been validated both in large cohorts as well as in a randomised trial and has a class 1 recommendation in the ESC guidelines [6, 20]. In the US, the slightly higher LoQ 0 h < 6 ng/L cutoff is recommended by the FDA. Our results, however, suggest that both these thresholds may be unnecessarily low and might lead to further testing and potential admissions. Using a 0 h hs-cTnT threshold of <9 ng/L in our cohort would have enabled a safe discharge of about 60% of the patients. Compared to the threshold of <5 ng/L, a 0 h hs-cTnT <9 ng/L would have resulted in a rapid discharge of about 24% more patients in absolute terms (35.8% vs. 59.7%, respectively) with a clinically insignificant change in NPV (99.8% vs. 99.6%, respectively). Our results are in line with the previous findings from Shah et al. who found that a data-driven higher cutoff than the LoD could be used with an hs-cTnI assay for safe and efficient rule out of AMI and cardiac death [10]. However, to our knowledge, this is the first study to establish such a cutoff for the hs-cTnT assay. Previous RCTs evaluating an hs-cTnT <5 ng/L or hs-cTnI <5 ng/L approach have shown that the adherence to using a single troponin rule-out strategy by ED physicians is good [8, 21].

The sensitivity of our derived cutoff was around 96% which may seem low. It is, however, the post-test probability that clinicians are interested in when managing chest pain patients, which is obtained from the NPV or LR-. A NPV ≥99.5% is, therefore, commonly used as a safety threshold in chest pain studies [10], and this threshold seems to be accepted by most ED physicians [19]. In clinical practice, a single TnT rule-out strategy will be applied on those with a 0 h hs-cTnT measured >3 h after symptom onset; there was no new ischemic ECG changes and a nonhigh risk history, and in this group, both the NPV and the sensitivity were 100%. For physicians who only want to use a cutoff with a sensitivity of >99%, only 0 h < 5 ng/L and not even the LoQ 0 h < 6 ng/L cutoff fulfilled this goal. The 2021 guidelines for management of chest pain also state that low-risk patients should be defined as those having a <1% risk of having a 30-day MACE [1]. The NPV obtained in this study confers a 30-day risk of AMI/death of only 0.4% and is thereby well within this safety threshold. In addition, the sensitivity of our cutoff was similar to that seen with other rule-out pathways such as using hs-cTnI <5 ng/L with the High-STEACS algorithm. This hs-cTnI <5 ng/L strategy has had a sensitivity of 94.5–97.1% in different cohorts [22, 23], yet in a large RCT, it was shown to be safe [21]. In addition, we had no data on the time between the onset of chest pain and hs-cTnT testing in the derivation cohort and validation cohort 1, and by not excluding those with a 0 h hs-cTnT measurement ≤3 h after symptom onset where current guidelines recommend a second hs-cTnT measurement [6], our NPVs and sensitivity metrics may thereby be falsely too low. Although this comes at the cost of a slightly decrease in the proportion of ruled-out patients, this will probably still be more effective than the corresponding <5 or <6 ng/L strategies.

The subgroup analyses demonstrated relatively uniform results across clinically relevant subgroups, but the NPV for patients with a history of AMI was below our predefined threshold of ≥99.5%, which is not surprising since this is a high-risk group with a high pretest probability of AMI. Likewise, the NPV also seemed to be somewhat lower in those ≥65 years and those with renal glomerular filtration rate <60 ml/min/1.73 m^2^. The upper CI did, however, include 99.5% and the confidence intervals were somewhat wide, and this needs further evaluation in other studies. In clinical practice, the NPVs will likely be even higher since clinicians also incorporate other clinical parameters such as symptoms and ECG in the decision making [12].

A favourable long-term prognosis is a less relevant factor for the decision of discharging a chest pain patient from the ED but supports the safety of a rule-out strategy. It is, therefore, reassuring that the cutoff <9 ng/L identifies not only a very low short-term risk in the ED chest pain patients but also a low 1-year mortality of only 0.6–0.7% in both study cohorts. This is similar to the 1-year cardiac death rates (0.9%) seen in patients randomised to discharge using the High-Sensitivity Troponin in the Evaluation of Patients with Acute Coronary Syndrome (High-STEACS) algorithm [21].

### 4.1. Strengths and Limitations

We evaluated a large cohort of ED chest pain patients included from different hospitals of different sizes and outcomes were ascertained by national registries with excellent coverage and accuracy. Our results were validated in a large external ED cohort, increasing their credibility and generalisability.

First, although we included chest pain patients from different EDs of different sizes, all hospitals were in Sweden with an AMI/death prevalence reflecting what is commonly seen in Swedish EDs [7]. This prevalence is similar to the prevalence of about 5% seen in US EDs [1] but lower than what is seen in some European centers [3]. The performance of this new 0 h hs-cTnT strategy may thereby differ in settings with a higher AMI/death prevalence. However, the LR(-) of 0.06 indicates that the hs-cTnT threshold of <9 ng/L will identify patients with a very low risk also in settings with a higher prevalence, especially when combined with the clinician's assessment of patient history and ECG. We also provide NPVs and LR for all cutoffs, and in settings where a risk >0.5% is considered acceptable, an even higher cutoff may be used which would identify even more patients for a safe and early rule out.

Second, an hs-cTnT below the threshold should never replace clinical judgement and a holistic approach to the individual patient. The slightly lower NPV among patients with previous AMI is an indication of this. We did not have data on the physician's assessment of the clinical history and ECG, and the effects of adding this information are, therefore, unclear. However, considering what has been seen in other studies, it will likely increase the NPV further and slightly decrease the proportion of ruled-out patients, but this will probably still be more effective than the corresponding <5 or <6 ng/L strategies [12].

Third, we have no data on the time between the onset of chest pain and hs-cTnT testing which is relevant due to the kinetics of troponin release. However, when combined with a normal ECG, the performance of the LoD strategy did not differ in early presenters in the LoDeD RCT, and the LoD strategy has also been shown to perform well in those presenting as early as 1 h after symptom onset [8, 24]. We do, however, believe that this needs further evaluation in other studies.

Fourth, both cohorts were observational and not managed in accordance with our derived cutoff. The true effects on safety and efficacy if implemented are, therefore, unknown. Previous studies on the hs-cTnT <5 ng/L or <6 ng/L strategies are, however, except for one RCT with 639 patients, also based exclusively on observational data. In this regard, it should be noted that our study included more patients than all previous studies evaluating those strategies combined. The confidence intervals were narrow and consistent across the two large cohorts, which support the safety of this approach. The use of our Swedish national registries also enabled complete follow-up in 99.99% of the patients. Consequently, our results need to be further confirmed in other settings and preferentially in a randomised trial comparing a rule-out strategy using <5 ng/L and the threshold in the present study of <9 ng/L.

Fifth, we did not use adjudicated diagnoses of AMI but instead relied on diagnoses from our national Swedish registries. These registries have, however, been shown to have excellent coverage as well as accuracy [14, 16]. We have also previously shown that the agreement between these diagnoses and adjudicated diagnoses is high [25].

## 5. Conclusions

A higher 0 h hs-cTnT cutoff of <9 ng/L safely rules out AMI/death within 30 days in most chest pain patients and is likely a more effective strategy than the currently recommended <5 ng/L or <6 ng/L cutoffs. The use of this new higher cutoff has the potential to improve management of ED chest pain patients.

## Figures and Tables

**Table 1:** Baseline patient characteristics.

Total	Derivation cohort	Validation cohort 1	Validation cohort 2
	*N* = 24973	*N* = 132021	*N* = 1138
Demographics			
Age (y)	61.0 (46.0–74.0)	56.6 (41.8–70.8)	63.1 (48.9–73.7)
Men	12957 (51.9%)	67841 (51.4%)	638 (54.7%)
Women	12016 (48.1%)	64180 (48.6%)	529 (45.3%)
Arrival by ambulance	8662 (34.7%)	30741 (23.2%)	476 (40.8%)
Past medical history			
Hypertension	5666 (22.7%)	29050 (22%)	506 (43.4%)
Hyperlipidemia	1282 (5.1%)	12012 (9.1%)	262 (22.5%)
Diabetes	3535 (14.2%)	11195 (8.4%)	162 (13.9%)
History of CAD	3924 (15.7%)	13658 (10.3%)	333 (28.5%)
History of AMI	2647 (10.6%)	9831 (7.5%)	235 (20.1%)
History of stroke/TIA	1812 (7.3%)	5245 (4.0%)	105 (9.0%)
COPD	1058 (4.2%)	4384 (3.3%)	88 (7.5%)
In-hospital characteristics			
GFR <60 mL/min/1.73 m^2^	3172 (12.7%)	15001 (11.4%)	176 (15.1%)
Time to hs-cTnT sample <60 min	19444 (77.9%)	107077 (81.1%)	NA
Time to hs-cTnT sampling (min)	34.0 (20–57)	26 (13–48)	NA
Medications at presentation			
ACE-I/ARB	6323 (25.3%)	32341 (24.5%)	364 (31.2%)
Aspirin	4261 (17.1%)	22526 (17.1%)	351 (30.1%)
Statin	5578 (22.3%)	24120 (18.3%)	349 (29.9%)
Beta-blocker	3380 (13.5%)	30363 (23.0%)	355 (30.4%)
Diuretics	4091 (16.4%)	15749 (11.9%)	239 (20.5%)
Nitroglycerine	1551 (6.2%)	8889 (6.7%)	269 (23.1%)
NOAC/Warfarin	1736 (6.9%)	8351 (6.3%)	116 (9.9%)
P2Y12 inhibitor	931 (3.7%)	3658 (2.8%)	81 (6.9%)
Outcomes			
AMI/death within 30 days	1668 (6.7%)	7668 (5.8%)	93 (8.0%)
AMI 30 days	1458 (5.8%)	6924 (5.2%)	89 (7.6%)
Death within 30 days	264 (1.1%)	1045 (0.8%)	5 (0.4%)
Death within 365 days	1210 (4.8%)	4888 (3.7%)	NA

Values are the median (25th percentile and 75th percentile) or *n* (%). ACEi: ACE inhibitors; AMI: acute myocardial infarction; ARB: angiotensin receptor blockers; CAD: coronary artery disease; COPD: chronic obstructive pulmonary disease; GFR: glomerular filtration rate (MDRD 4); Hs-cTnT: high-sensitive cardiac troponin T; NOAC: novel oral anticoagulants; STEMI: ST-elevation myocardial infarction; TIA: transient ischemic attack.

## Data Availability

The data used to support the findings of this study are available on request from the corresponding author.

## Supplementary Materials

Supplementary Materials Appendix 1: data collection validation cohort 1. Appendix 2: characteristics of the hospitals included in the study. Appendix 3: adjudication in the derivation cohort. Appendix 4: negative predictive value (NPV) and likelihood ratio (LR) for different 0 h hs-cTnT levels in the derivation cohort for ruling out AMI or all-cause death within 30 days. Appendix 5: cumulative incidence of 1-year all-cause mortality in validation cohort 1 stratified according to 0 h hs-cTnT concentrations. Appendix 6: supplemental table 2: negative predictive value (NPV) and likelihood ratio (LR) in validation cohort 2 for ruling out AMI or all-cause death within 30 days.
