# Supplementary material for: A Single High-Sensitivity Cardiac Troponin T Strategy for Ruling Out Myocardial Infarction
Source: Emerg Med Int. 2024 Mar 25;2024:2241528. doi: 10.1155/2024/2241528 (PMC10985641; doi:10.1155/2024/2241528)
Supplement: Supplementary Materials — Appendix 1: data collection validation cohort 1. Appendix 2: characteristics of the hospitals included in the study. Appendix 3: adjudication in the derivation cohort. Appendix 4: negative predictive value (NPV) and likelihood ratio (LR) for different 0 h hs-cTnT levels in the derivation cohort for ruling out AMI or all-cause death within 30 days. Appendix 5: cumulative incidence of 1-year all-cause mortality in validation cohort 1 stratified according to 0 h hs-cTnT concentrations. Appendix 6: supplemental table 2: negative predictive value (NPV) and likelihood ratio (LR) in validation cohort 2 for ruling out AMI or all-cause death within 30 days. [file 2241528.f1.docx]

**Supplemental material**

**Appendix 1**

**Data collection validation cohort 1**

Information on all patient visits to the ED were retrieved from the hospitals’ local administrative databases, and laboratory data was obtained from the hospitals’ IT Departments. At all sites, the Elecsys 2010 system (Roche Diagnostics, Mannheim, Germany) was used to analyze hs-cTnT levels. Data on comorbidities and use of medication was obtained from the National Patient Register and the Prescribed Drug Register, respectively. Comorbidities were classified according to all discharge diagnoses from all hospital contacts before the index hospitalization, and ongoing medication usage was defined as ≥2 dispensed prescriptions during the year preceding the index date.

Diagnoses at the index visit were based on the primary discharge diagnosis, coded in the National Patient Register according to ICD-10. An acute MI diagnosis was defined as ICD-codes I21 or I22 in the National Patient Register, and data on all-cause mortality was obtained from the Cause of Death register. Both registries provide complete nationwide coverage.

**Appendix 2**

**Characteristics of the hospitals included in the study**

Patients in the validation cohort 1 were included from the following hospitals in Sweden:

I) Karolinska University Hospital, Solna, Stockholm, and Karolinska University Hospital Huddinge, Stockholm: Karolinska University Hospital is located at two sites, in Solna and Huddinge. Karolinska University Hospital Solna is situated in the northern part of Stockholm city and has a yearly attendance at the ED of approximately 74,000. The second site is located south of Stockholm in Huddinge municipality, which is administered by Stockholm County Council. The annual number of ED visits in Huddinge is approximately 75,000. During the study period, on-site coronary angiography and PCI were only available at Huddinge during ofﬁce hours, but at all times at Karolinska University Hospital Solna.

II) Danderyds Hospital: Danderyds Hospital is one of the largest emergency hospitals in Sweden, located in the northern parts of Stockholm city. The hospital had approximately 95,000 visits per year to the ED during the study period, and onsite-PCI available 24/7.

III) St. Görans Hospital, Stockholm: St Görans Hospital is centrally located in Stockholm city and had approximately 77,000 annual visits to the ED during the study period. At the time, on-site coronary angiography and PCI were only available during office hours.

IV) Stockholm South General Hospital, Stockholm: Stockholm South General Hospital is located in the southern part of Stockholm city and has the largest emergency care unit in the Nordic region. The annual number of visits to the ED is approximately 120,000. At the time of the study, coronary angiography was only available during office hours.

V) Södertälje Hospital, Stockholm: Södertälje Hospital is located outside Stockholm city, in Södertälje municipality, but this is administered by Stockholm County Council. The site has the lowest yearly ED attendance (approximately 32,000) of the hospitals included in the cohort. Södertälje Hospital is the only hospital within Stockholm County that did not provide coronary angiography on-site during the study period. Therefore, patients in whom coronary angiography were indicated were transferred to one of the other hospitals in Stockholm.

VI) Sahlgrenska University Hospital, Sahlgrenska, Östra and Mölndal, Gothenburg:

The three hospitals were merged in 1997. All sites are located in the city of Gothenburg, and the hospital has a capacity of 2,000 beds in total. Altogether, there are approximately 106,000 visits per year to the adult EDs.

Patients in the Derivation cohort and validation cohort 2 were included from the following hospitals in Sweden:

VII) Skane University Hospital in Lund: The ED annual census is about 65000, of which 5500 visits have a primary complaint of chest pain, and there is PCI lab availability 24/7.

VIII) Helsingborg Hospital: The ED annual census is about 90000 with 5000 annual visits due to chest pain, and there is a cath lab available during office hours, with STEMI patients going to Skane University Hospital in Lund outside office hours.

VIII) Ystad Hospital: the ED annual census is about 30000, of which 2000 have a primary complaint of chest pain, and there is no PCI lab; STEMI patients are transported to Skane University Hospital in Lund.

X) Skane University Hospital in Malmö: Annual ED census of about 90000 with 7000 yearly visits due to chest pain. There is a cath lab available during office hours, with STEMI patients going to Skane University Hospital in Lund outside office hours.

XI) Kristianstad hospital: Annual ED census of about 55000, with 3500 visits due to chest pain. There is a cath lab available during office hours, with STEMI patients going to Skane University Hospital in Lund the remaining time.

**Appendix 3**

**Adjudication in the derivation cohort**

Patients discharged from the ED with any of the following ICD-codes/procedure codes during the 30-day follow-up underwent adjudication:

**ICD-codes**

| I46.9 | Cardiac arrest, cause unspecified |
| --- | --- |
| I46.0 | Cardiac arrest with successful resuscitation |
| R57.0 | Cardiogenic shock |
| I47.2 | Ventricular tachycardia |
| I49.0 | Ventricular fibrillation and flutter |
| I44.2 | Atrioventricular block, complete |
| I44.1B | Atrioventricular block, second degree |
| I21.0 | Acute transmural myocardial infarction of anterior wall |
| I21.1 | Acute transmural myocardial infarction of inferior wall |
| I21.2 | Acute transmural myocardial infarction of other sites |
| I21.3 | Acute transmural myocardial infarction of unspecified site |
| I21.4 | Acute subendocardial myocardial infarction |
| I21.4A | Acute subendocardial myocardial infarction of anterior wall |
| I21.4B | Acute subendocardial myocardial infarction of inferior wall |
| I21.4W | Acute subendocardial myocardial infarction of other sites |
| I21.4X | Acute subendocardial myocardial infarction of unspecified site |
| I21.9 | Acute myocardial infarction, unspecified |
| I22.0 | Subsequent ST elevation myocardial infarction of anterior wall |
| I22.1 | Subsequent ST elevation myocardial infarction of inferior wall |
| I22.8 | Subsequent ST elevation myocardial infarction of other sites |
| I22.9 | Subsequent ST elevation myocardial infarction of unspecified site |
| I20.0 | Unstable angina |
| I24.9 | Acute ischemic heart disease, unspecified |

**Procedure codes**

| FNA,FNB,FNC,FND,FNE,FNF or FNG | Revascularization (PCI or CABG) |
| --- | --- |
| DF005 | Intra-Aortic Balloon Pump Therapy |
| DF013 | External pacemaker treatment |
| TFP00 | Temporary transvenous or epicardial pacing |
| ZXG40 | Transvenous pacemaker implantation |
| FPE20 | Transvenous pacemaker with atrial and ventricle leads implantation |
| FPE00 | Transvenous pacemaker with ventricle lead implantation |
| FPE26 | Transvenous biventricular pacemaker implantation |
| FPE10 | Transvenous pacemaker with atrial lead implantation |
| DF025 | Cardioversion/defibrillation due to ventricular arrythmia. |
| DF012 | Chest compressions |
| DF017 | Mechanical chest compressions |
| DF028 | Cardiopulmonary resuscitation (CPR) |

**Appendix 4**

Negative predictive value (NPV) and Likelihood ratio (LR) for different 0h hs-cTnT levels in the derivation cohort for ruling out AMI or all-cause death within 30-days.

| **Hs-cTnT (ng/L)** | **NPV**  **(95%CI)** | **Sensitivity**  **(95%CI)** | **Specificity**  **(95%CI)** | **LR(+)**  **(95%CI)** | **LR(-)**  **(95%CI)** |
| --- | --- | --- | --- | --- | --- |
| <5 | 99.8 (99.7–99.9) | 99.2 (98.6–99.5) | 38.3 (37.7–39.0) | 1.6 (1.6–1.6) | 0.02 (0.01–0.04) |
| <6 | 99.8 (99.7–99.8) | 98.4 (97.7–99.0) | 46.9 (46.2–47.5) | 1.9 (1.8–1.9) | 0.03 (0.02–0.05) |
| <7 | 99.7 (99.6–99.8) | 97.7 (96.8–98.3) | 54.0 (53.3–54.6) | 2.1 (2.1–2.2) | 0.04 (0.03–0.06) |
| <8 | 99.6 (99.5–99.7) | 96.8 (95.9–97.6) | 59.4 (58.7–60.0) | 2.4 (2.3–2.4) | 0.05 (0.04–0.07) |
| <9 | 99.6 (99.5–99.7) | 96.2 (95.2–97.1) | 63.7 (63.1–64.3) | 2.7 (2.6–2.7) | 0.06 (0.05–0.08) |
| <10 | 99.4 (99.3–99.5) | 94.6 (93.4–95.6) | 67.4 (66.8–68.0) | 2.9 (2.8–3.0) | 0.08 (0.07–0.10) |
| <11 | 99.3 (99.2–99.5) | 93.4 (92.1–94.6) | 70.5 (69.9–71.1) | 3.2 (3.1–3.2) | 0.09 (0.08–0.11) |
| <12 | 99.2 (99.1–99.4) | 92.2 (90.8–93.5) | 73.2 (72.6–73.76) | 3.4 (3.4–3.5) | 0.11 (0.09–0.13) |
| <13 | 99.1 (99.0–99.3) | 90.7 (89.2–92.1) | 75.4 (74.9–76.0) | 3.7 (3.6–3.8) | 0.12 (0.11–0.14) |
| <14 | 99.1 (98.9–99.2) | 89.7 (88.1–91.1) | 77.5 (76.9–78.0) | 4.0 (3.9–4.1) | 0.13 (0.12–0.15) |
| <15 | 99.0 (98.8–99.1) | 88.4 (86.7–89.9) | 79.3 (78.8–79.8) | 4.3 (4.1–4.4) | 0.15 (0.13–0.17) |

**Appendix 5**

Cumulative incidence of 1-year all-cause mortality in the validation cohort 1 stratified according to 0h hs-cTnT concentrations.


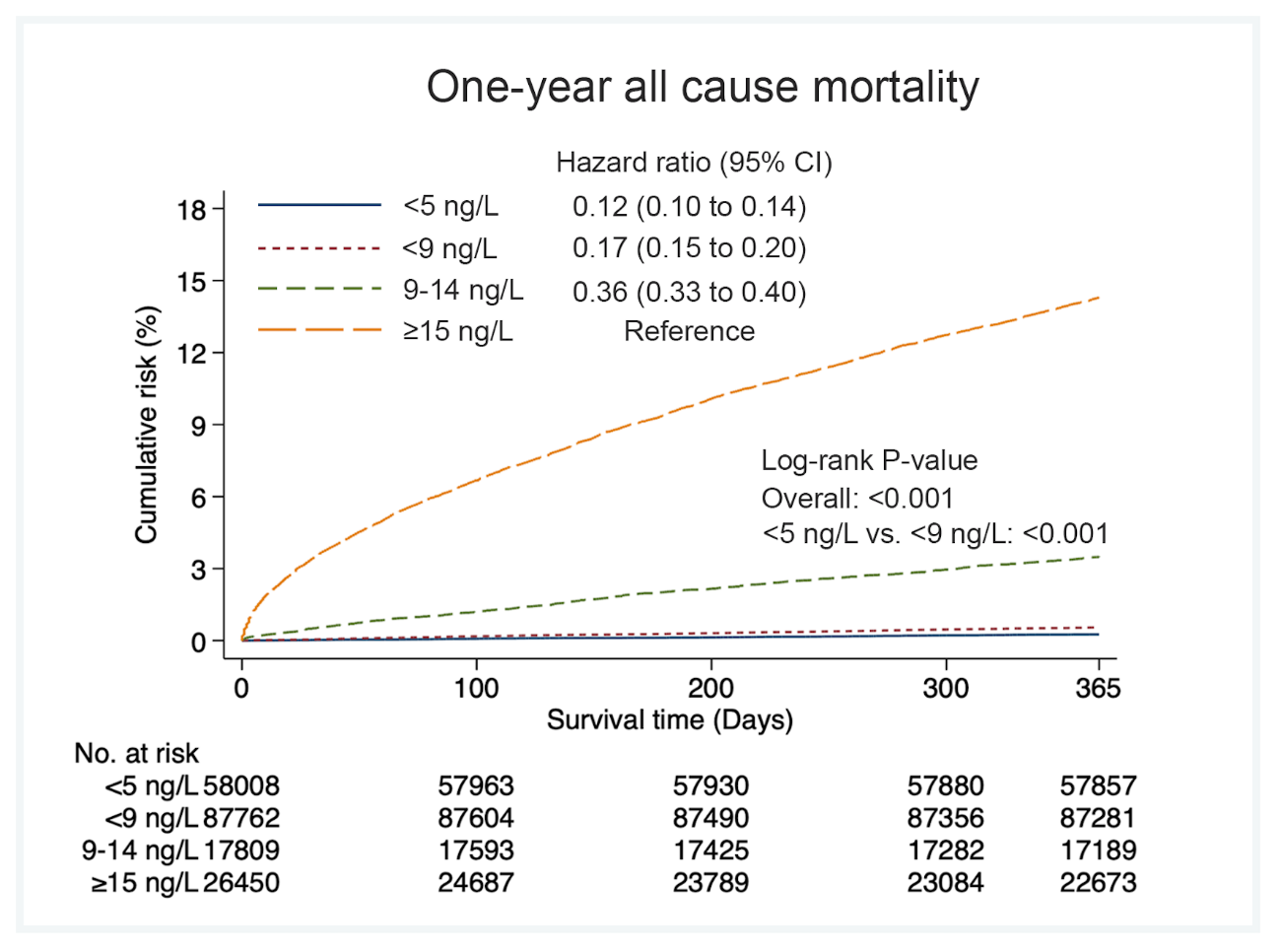


**Appendix 6**

**Supplemental table 2**

Negative predictive value (NPV) and Likelihood ratio (LR) in the validation cohort 2 for ruling out AMI or all-cause death within 30-days.

| **Hs-cTnT (ng/L)** | **NPV**  **(95%CI)** | **Sensitivity**  **(95%CI)** | **Specificity**  **(95%CI)** | **LR(+)**  **(95%CI)** | **LR(-)**  **(95%CI)** |
| --- | --- | --- | --- | --- | --- |
| <5 | 99.4 (98.0–99.9) | 97.9 (92.5–99.7) | 32.5 (29.7–35.4) | 1.5 (1.4–1.5) | 0.07 (0.02–0.26) |
| <6 | 99.6 (98.5–100.0) | 97.9 (92.5–99.7) | 42.9 (39.9–46.0) | 1.7 (1.6–1.8) | 0.05 (0.01–0.20) |
| <7 | 99.3 (98.2–99.8) | 95.7 (89.4–98.8) | 50.7 (47.7–53.8) | 1.9 (1.8–2.1) | 0.08 (0.03–0.22) |
| <8 | 99.4 (98.3–99.8) | 95.7 (89.4–98.8) | 56.6 (53.6–59.6) | 2.2 (2.0–2.4) | 0.08 (0.03–0.20) |
| <9 | 99.4 (98.5–99.8) | 95.7 (89.4–98.8) | 61.9 (58.9–64.8) | 2.5 (2.3–2.7) | 0.07 (0.03–0.18) |
| <10 | 99.3 (98.4–99.8) | 94.6 (87.9–98.2) | 66.0 (63.1–68.9) | 2.8 (2.5–3.1) | 0.08 (0.03–0.19) |
| <11 | 99.3 (98.5–99.8) | 94.6 (87.9–98.2) | 69.9 (67.1–72.7) | 3.2 (2.8–3.5) | 0.08 (0.03–0.18) |
| <12 | 99.1 (98.2–99.6) | 92.5 (85.1–96.9) | 72.8 (70.0–75.5) | 3.4 (3.0–3.8) | 0.10 (0.05–0.21) |
| <13 | 99.0 (98.1–99.6) | 91.4 (83.8–96.2) | 74.5 (71.8–77.1) | 3.6 (3.2–4.0) | 0.12 (0.06–0.22) |
| <14 | 98.7 (97.7–99.3) | 88.2 (79.8–94.0) | 77.0 (74.4–79.5) | 3.8 (3.4–4.4) | 0.15 (0.09–0.27) |
| <15 | 98.6 (97.6–99.3) | 87.1 (78.6–93.2) | 79.0 (76.4–81.4) | 4.1 (3.6–4.8) | 0.16 (0.10–0.28) |
